# When Does Diversity Trump Ability (and Vice Versa) in Group Decision Making? A Simulation Study

**DOI:** 10.1371/journal.pone.0031043

**Published:** 2012-02-16

**Authors:** Shenghua Luan, Konstantinos V. Katsikopoulos, Torsten Reimer

**Affiliations:** 1 Center for Adaptive Behavior and Cognition, Max Planck Institute for Human Development, Lentzeallee 94, Berlin, Germany; 2 Center for Adaptive Behavior and Cognition, Max Planck Institute for Human Development, Berlin, Germany; 3 Department of Communication, Purdue University, West Lafayette, Indiana, United States of America; Queen Mary, University of London, United Kingdom

## Abstract

It is often unclear which factor plays a more critical role in determining a group's performance: the diversity among members of the group or their individual abilities. In this study, we addressed this “diversity vs. ability” issue in a decision-making task. We conducted three simulation studies in which we manipulated agents' individual ability (or accuracy, in the context of our investigation) and group diversity by varying (1) the heuristics agents used to search task-relevant information (i.e., cues); (2) the size of their groups; (3) how much they had learned about a good cue search order; and (4) the magnitude of errors in the information they searched. In each study, we found that a manipulation reducing agents' individual accuracy simultaneously increased their group's diversity, leading to a conflict between the two. These conflicts enabled us to identify certain conditions under which diversity trumps individual accuracy, and vice versa. Specifically, we found that individual accuracy is more important in task environments in which cues differ greatly in the quality of their information, and diversity matters more when such differences are relatively small. Changing the size of a group and the amount of learning by an agent had a limited impact on this general effect of task environment. Furthermore, we found that a group achieves its highest accuracy when there is an intermediate amount of errors in the cue information, regardless of the environment and the heuristic used, an effect that we believe has not been previously reported and warrants further investigation.

## Introduction

Scientists from a variety of disciplines have studied extensively the abilities of humans and animals to solve problems in groups [1]–[4]. Of the factors identified as affecting a group's performance, two appear especially important: the diversity among members of a group and their individual abilities [3], [5]–[7]. However, the contributions of these two factors to group performance are not always equal. In judgment tasks where the value of a continuous variable of interest needs to be estimated, diversity has been shown to matter more than individual ability [4], [5], [8]; in problem-solving tasks where the problems are technical or mathematical in nature and difficult to solve, the reverse tends to be true [8]–[10]. In other tasks, such as decision-making and creative problem solving, it is often unclear which factor plays a more critical role [3], [10]–[12]. In this study, we tried to address this “diversity vs. ability” issue in a decision-making task. Through three simulation studies in which we systematically manipulated variables affecting both factors, we identified conditions when diversity trumps ability, and vice versa.

In the type of decision problems probed in our study, an agent or a group of agents was asked to decide which of two options (e.g., two patches) had a larger value on a certain criterion (e.g., amount of food) on the basis of some relevant cues (e.g., smell, visual pattern, etc.). Of the possible individual strategies applicable to make this decision, we implemented two in this study that belong to the family of “fast and frugal” heuristics [13]–[15]: take-the-best and minimalist (see details of their algorithms in Methods and Analyses). They are heuristics because they do not attempt to search for all the available cues and integrate information from all searched cues to make a decision. Instead, they search cues sequentially and employ a simple “one-reason” decision-making mechanism; that is, search stops as soon as a difference between two options on a cue is found and a decision is made according to their values on this stopping cue alone. The two heuristics differ in their search rules: Whereas take-the-best searches cues in the order of their *validities*, a measure of cue information quality, minimalist searches cues randomly.

We focused on take-the-best and minimalist in this study for two reasons. First, heuristics with structures identical or similar to those of take-the-best and minimalist have been found to be adopted by both humans and other species [15]–[19]. For example, when choosing between two mating candidates, female guppies, *Poecilia reticulata*, often rely on two cues: the amount of orange body color (a genetic cue) and whether another female has mated with the candidate before (a social cue). A female guppy will use the social cue only when the difference between two males on the genetic cue is not large enough (<40%) [18]. In another example, when deciding which flower of two matches a model flower for foraging, honeybees usually check cues in the order of odor, color, and shape, using a latter cue only when the earlier cue or cues fail to distinguish [19]. The mating strategy of female guppies cannot be easily extended to a group context, but this can be done for the foraging strategy of honeybees: Each individual bee in a colony may adopt a strategy similar to the one just described, and then they may apply a majority or quorum rule to make a group decision where to forage [20].

The second reason why we focused on take-the-best and minimalist is that their different search rules enable us to examine the relative contributions of diversity and ability in group decision-making. Specifically, in the context of our study, ability was represented by the average decision accuracy achieved by individual agents in a group, and diversity was captured by the range of information searched and the subsequent decisions made by their group collectively. The ability of a group of agents using take-the-best (the take-the-best group) tends to be higher than that of a group of minimalist agents (the minimalist group), due to the better search rule implemented in take-the-best. However, the diversity level of the former is typically lower than that of the latter, because searching cues randomly usually leads each minimalist agent to search a different set of cues, increasing the collective cue set explored by the group.

The contrasts between the take-the-best and minimalist groups formed the basis of our investigation. In three studies, we examined groups' performances in four task environments that differed in the distribution of cues' validities (see Methods and Analyses), which has been found as an ecological variable critical to the individual decision accuracies of the take-the-best and minimalist agents [21], [22]. On the platform of these four task environments, we compared: (1) take-the-best and minimalist groups with varying group sizes; (2) take-the-best groups with agents who had different amounts of learning regarding the cue validity order; and (3) groups, both take-the-best and minimalist, with agents who made decisions based on cue information with varied degrees of errors. All of the three variables, group size, individual learning, and information errors, can directly affect individual accuracy and/or diversity and indirectly affect their contributions to group decision accuracy. Finally, after each agent within a group made an individual decision, a simple majority rule was applied to determine the group decision. Although it is a popular aggregation rule adopted by many groups in realistic settings [20], [23], [24], the rule itself does not explicitly consider the possible communications among group members and how the information or decisions by others (i.e., public information) may affect the quality of group decisions [4], [25], [26].

## Results and Discussion

### Study 1: Group Size

**Figure 1:**
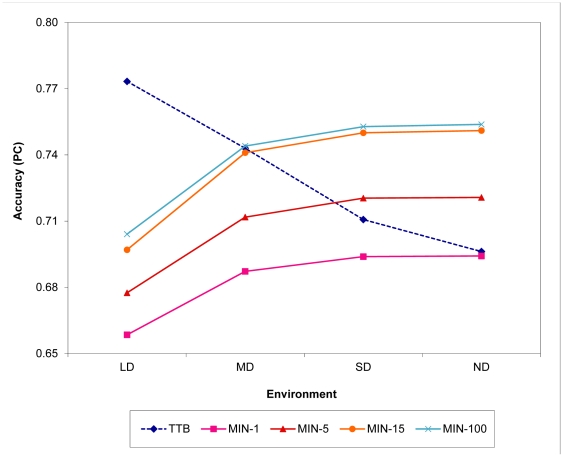
Results from Study 1: group size. Note that there was no effect of group size on a take-the-best (TTB) group's performance in this set of simulations, because the group was assumed to be totally homogeneous. MIN-1, MIN-5, MIN-15, and MIN-100 stand for a minimalist group with 1, 5, 15, and 100 agents, respectively. Environments differed in their distribution of cue validities: LD, large difference; MD, medium difference; SD, small difference; ND, no difference. PC: Percentage correct.

In this set of simulations, we pitted a take-the-best group directly against a minimalist group, with group size *m* ranging from 1 to 100. Here, we assumed that all agents in the take-the-best group searched cues in the same order, the order according to cues' objective validities, and the information from each cue was error free. In this way, the individual accuracy of a take-the-best agent was at its maximum, but the diversity of the take-the-best group was at its minimum—zero—because all take-the-best agents searched the same cues and made the same decisions. Consequently, there was no difference between the decisions of a take-the-best agent and those of a take-the-best group; thus, group size had no effect on this group's decision accuracy. In contrast, a larger group size could still increase the diversity level of a minimalist group (see File S1 for the diversity results) and affect its decision accuracy positively. The results of this study are shown in Figure 1.

Three main results can be seen from Figure 1. First, the minimalist group, which had a higher level of diversity but a lower level of individual accuracy, outperformed the take-the-best group, which had the opposite characteristics, in task environments where the distribution of cue validities was relatively flat (e.g., the small-difference, SD, environment) and when group size was moderately large (*m*>4). Second, it was very difficult for a minimalist group to beat a take-the-best group when cues differed drastically in their validities. In the large-difference (LD) environment, for instance, the accuracy of a minimalist group with 100 agents still lagged far behind that of a take-the-best group, which was equivalent to the accuracy of one take-the-best agent. Third, group size had a positive effect on the accuracy of minimalist groups, but the effect became increasingly smaller when group size got larger. The pool of information (e.g., number of cues) from which agents draw their decisions constrained the effect of group size: The more scarce the information, the less the effect, and the fewer members will a group need to reach the highest level of accuracy [6].

Overall, the results in this study show that the relative importance of group diversity and individual accuracy depends on the informational characteristics of the task environment. When information is more evenly distributed among the cues, searching as many cues as possible by a group, which often depends on the size of the group and is a direct outcome of group diversity, will be more critical to the group's decision accuracy than agents' ability to make accurate decisions on their own. However, when the quality of the cues' information differs significantly, agents' knowledge of such differences, which results in higher individual accuracy, will matter more than the diversity of their group.

### Study 2: Individual Learning

Although the extreme uniformity of the take-the-best group in Study 1 was the logical outcome of the two assumptions made in that study, it is questionable whether these assumptions hold in reality. Let us start with cue order. Experiments with human participants have shown that different take-the-best users often search cues in different orders [27], [28]. Such variance is partly due to the varied experiences people have with the cues that can affect their perceptions of cues' validities. To reflect such individual differences, we incorporated a learning component for the take-the-best agents in this set of simulations (see details in Methods and Analyses).

**Figure 2:**
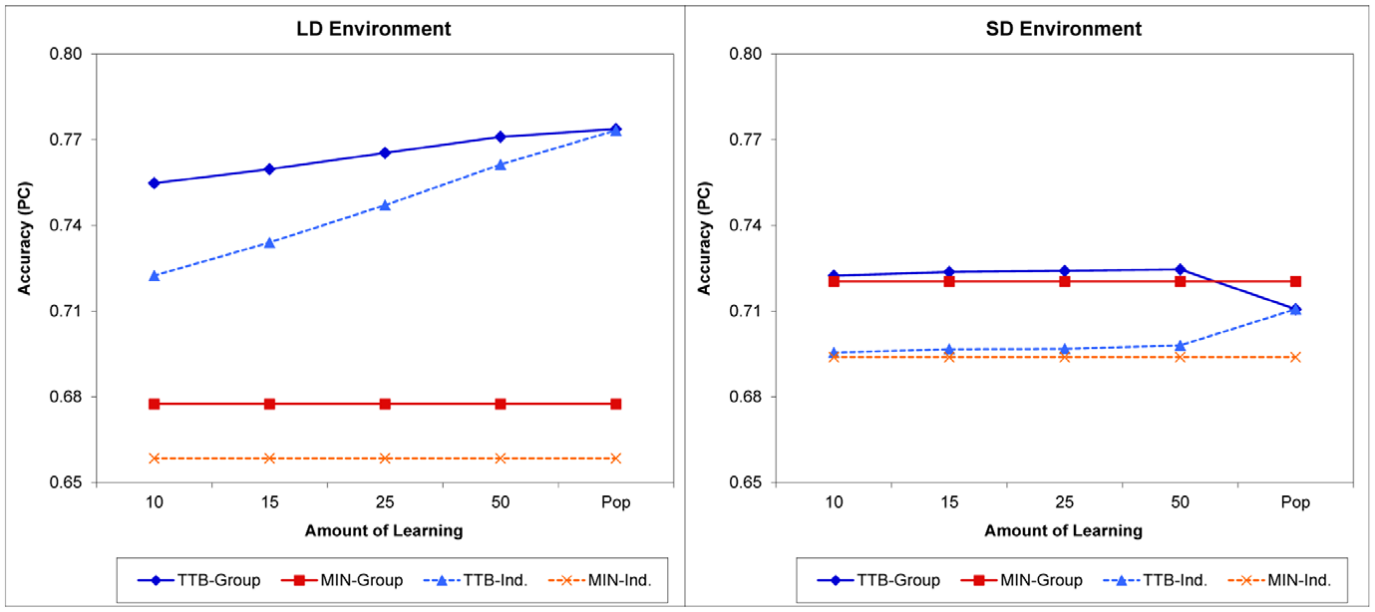
Results from Study 2: individual learning. Because the results in the MD and ND environments were similar to the results in the LD and SD environments, respectively, only results from the LD and SD environments are shown for the sake of brevity. Group size was 5 for all groups from which the results were derived, and the amount of learning was measured by the number of options in a learning sample. The lines for minimalist agents and groups are flat because no learning was assumed to take place for a minimalist agent in this set of simulations. Pop: Population. MIN-Group and MIN-Ind.: Minimalist group and individual agents. TTB-Group and TTB-Ind.: Take-the-best group and individual agents.

In general, each take-the-best agent started without knowledge of cues' validities and had to learn them through a randomly drawn learning sample. After this learning process, which typically resulted in agents learning different cue orders, a group of five agents were asked to make decisions on a common sample. We manipulated the number of options, *n*, in the learning sample experienced by each take-the-best agent. It varied from as small as 10 to as large as the entire population, in which all agents were assumed to eventually learn the objective cue validity order. The results of this study are illustrated in Figure 2. Because the results in the MD and ND environments were similar to the results in the LD and SD environments, respectively, for the sake of brevity, only results from the LD and SD environments are shown in Figure 2.

In the LD environment, one can see three major results (Figure 2). First, even a small amount of learning could help a take-the-best agent perform much better than a minimalist agent that was assumed to know nothing about cues' validities. Second, learning created diversity among take-the-best agents (see File S1 for the diversity results), which in turn created a positive difference between the accuracy of a take-the-best group and that of a single take-the-best agent in all but the “population” conditions. Third, a group of five take-the-best agents who knew less about cues' validity orders failed to beat one take-the-best agent who knew the order perfectly in this environment, as seen in the population condition. This shows again that in environments such as the LD in which cues differ greatly in their quality, it is essential for decision makers—either individuals or groups—to know which cues are informational and which are not and to search and use them accordingly.

The results in the SD environment differ from those in the LD environment. When learning was limited (from *n* = 10 to 50), a take-the-best agent always performed better than a minimalist agent, as did a take-the-best group over a minimalist group. Even though the two groups' performance differences were small, they were all in favor of the take-the-best group, which is in contrast to the result in Study 1 in the same environment (Figure 1). Why? Unlike in Study 1, there was diversity in the take-the-best group here. Furthermore, in the SD environment, because cues' validities were very close to each other, their differences in a learning sample were largely determined by random sampling errors. This made it difficult for take-the-best agents to learn the objective cue validity order and caused a rather flat learning curve with increased learning experience. However, for the same reason, it also became quite likely that one take-the-best agent would learn one cue order from its own sample while another agent would learn a different order from another, independent sample. This high level of diversity in cue order led to a high level of diversity in the information a take-the-best group searched for collectively. Combined with their higher individual accuracy, a group of learning take-the-best agents was now well equipped to outperform a group of minimalist agents in the SD environment.

With more learning, a take-the-best agent eventually achieved its best individual accuracy in the population condition; however, it is in this condition that the take-the-best group's performance dropped and became worse than that of the minimalist group once again. This occurred because the take-the-best agents, with the knowledge of the objective cue validity order, started to search uniformly again, and the whole group lost the diversity it once enjoyed when learning was limited. Such a loss of diversity was detrimental to the group's performance in environments like SD, where gathering a large quantity of information is more critical than knowing the quality of each piece of information.

### Study 3: Information Errors

Having addressed the issue of cue order through the control of individual learning, let us now turn to the quality of cue information. When agents search cues either in their memory or from the environment, there is no guarantee that the cue values they obtain will be perfectly accurate [29], [30]. When there are random errors in cue values, different agents may make different decisions for the same pair of options, even when they all adopt the same decision heuristic and search cues in the same order.

For instance, suppose that two flowers A and B are described by three cues: odor, color, and shape, and a cue value of “1” indicates flowers with better foraging values in terms of nectar content or pollen quality. Suppose further that A and B are with cue values of [1,0,1] and [1,1,0], respectively. Without information error, a take-the-best agent would stop search at the color cue and choose B. Meanwhile, if another take-the-best agent mistakenly perceives the color cue's value on B as “0” instead of “1,” it would stop search at the shape cue and choose A instead. Allowing for such errors in a task, it is possible that a group of take-the-best agents would stop search at different cues, leading them to make different decisions. On the other hand, the individual accuracy of these agents is likely to decrease because errors will reduce the quality of the cue information on which their decisions are based.

**Figure 3:**
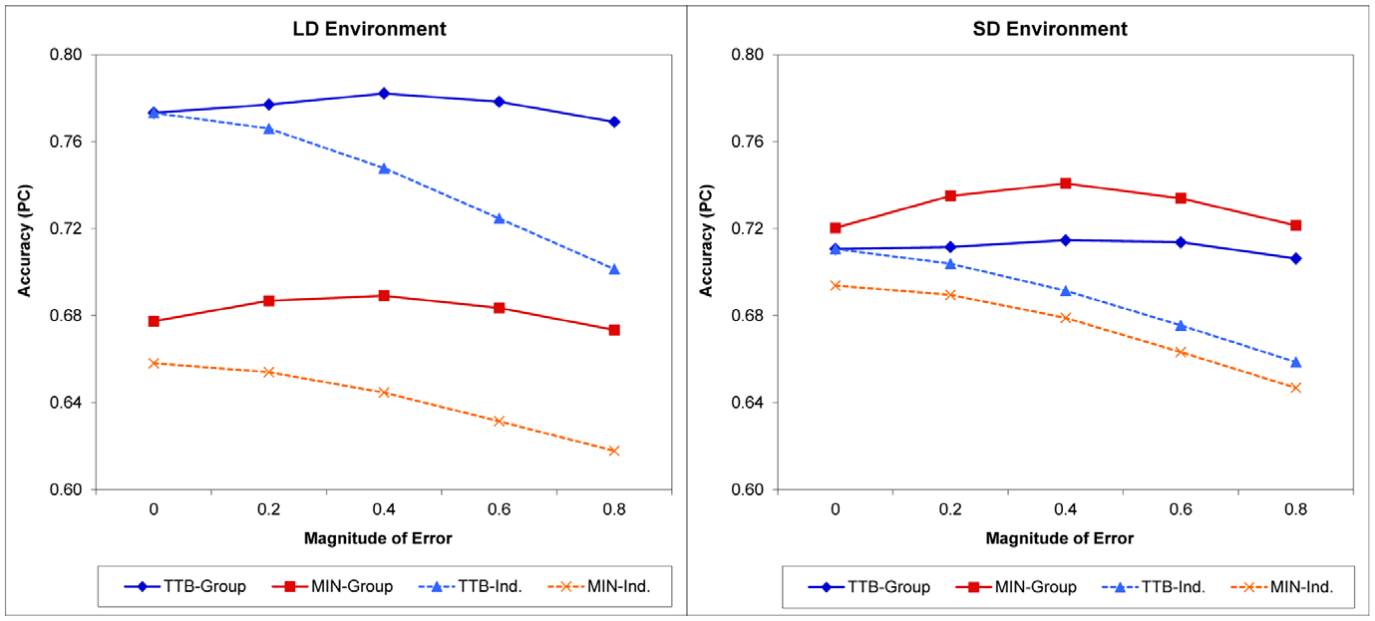
Results from Study 3: information errors. Because the results in the MD and ND environments were similar to the results in the LD and SD environments, respectively, only results from the LD and SD environments are shown for the sake of brevity. Group size was 5 for all groups from which the results were derived, and the magnitude of error was measured as the standard deviation of a normal distribution from which the random errors were generated. MIN-Group and MIN-Ind.: Minimalist group and individual agents. TTB-Group and TTB-Ind.: Take-the-best group and individual agents.

We created erroneous cue information by adding a random error component to the cues' true values (see details in Methods and Analyses). The magnitude of errors was controlled by σ, the standard deviation of the error distribution. Five levels of σ, 0, 0.2, 0.4, 0.6, and 0.8, were applied in this set of simulations, with a higher σ making it more likely that a cue's apparent value would deviate from its objective value. We investigated the effects of information errors on both types of agents and groups, take-the-best and minimalist, and the results are shown in Figure 3. Because the results in the MD and ND environments resembled those in the LD and SD environments, respectively, only results from the LD and SD environments are shown.

From Figure 3, we see that information errors were clearly detrimental to individual accuracy: As the error magnitude increased from σ = 0 to 0.8, both take-the-best and minimalist agents made less and less accurate decisions. However, the effect of these errors on group accuracy was not that straightforward. In all environments, the following general pattern emerged: With a larger error magnitude, group performance started to get *better*, reached its best at some intermediate error level, and only got worse slowly afterward. In other words, the right magnitude of information errors could actually help a group make better decisions than when there was no error. This rather counterintuitive result was observed no matter which heuristic, take-the-best or minimalist, agents were using and how cue validities were distributed. Why?

The reason, we speculate, is the two opposing effects random information errors had on group decisions. On the one hand, they undermined individual agents' performances by making cue values noisier and reducing the qualities of cues. This in turn reduced each agent's contribution to the group, dragging the group's performance down. On the other hand, those errors diversified the information searched by a group of agents and their individual decisions (see File S1 for the diversity results), and this increased diversity could push the group's performance up. With information errors in a task, these two opposing effects always existed but did not always cancel each other out perfectly. With smaller magnitudes of errors, the gain of group accuracy due to added diversity could compensate for the loss due to reduced individual accuracy, resulting in a net accuracy gain. As the magnitude of errors increased, the gain would reach its maximum at some intermediate level, with its exact value depending on factors such as the heuristic used, the cue validity distribution, and the size of the group. Finally, when there was too much error, the group accuracy gain disappeared, and groups would perform below the level they could achieve with no information error.

In addition to the finding that an intermediate amount of information errors can actually improve groups' decision accuracy, two other results deserve mention. First, group performance appeared quite robust against information errors. Even when errors were large and had severely reduced agents' individual accuracy (σ = 0.8), groups could still perform quite well compared to when there was no error (σ = 0). Second, information errors did not alter the general pattern of the take-the-best and minimalist groups' relative performances observed in Study 1. That is, a take-the-best group still performed better than a minimalist group in the LD environment and worse in the SD environment. Information errors did add diversity to both groups; however, as long as all take-the-best agents searched cues in the same order, their group would be less diverse than a minimalist group, making it difficult for the former to beat the latter in the SD environment.

## Discussion

Group diversity is often credited as the main reason for the remarkable intelligence and achievements demonstrated by human and animal groups [3], [31], [32]. Using computer simulations and mathematical analyses, Hong and Page [33] predicted that a group of randomly selected agents (the more diverse group) could in theory outperform a group of best-performing agents (the group with higher ability) in searching for the best solution to a problem (e.g., the design of a gasoline engine). In a study by Krause and colleagues [8], this “diversity-can-trump-ability” prediction was confirmed in one experiment in which human participants were asked to estimate the number of marbles in a jar, but not in another in which the same group of participants were asked to solve an abstract statistical problem. Following the footprints of these two studies, we tried to understand the roles of diversity and ability in a task of group decision-making instead of problem solving or number judgment, aiming to identify conditions when diversity can trump ability and vice versa through three simulation studies.

We found that a conflict between ability, represented by individual decision accuracy, and diversity was present in each of the three studies. In Study 1, it was a take-the-best group versus a minimalist group with varying group sizes; in Study 2, it was a group of take-the-best agents who knew the cue validity order outright versus another group of take-the-best agents who had to learn it; and in Study 3, it was one group of agents who made their decisions based on error-free information versus another group whose decisions were based on erroneous information. These conflicts indicate that it can be difficult to maintain high levels of diversity and ability within a group simultaneously—a manipulation augmenting one often ends up hurting the other [34], [35]—and show why trying to draw a definite conclusion of this “diversity vs. ability” debate could be a futile pursuit.

Controlling and exploring the effects of four task variables, we identified some conditions under which a more diverse group, despite having a lower level of individual accuracy, made more accurate group decisions than another group with the opposite characteristics, and vice versa. In general, we found that the informational characteristics of a task environment—the distribution of cues' validities in particular for the task focused on in this study—play a critical role in determining the winner of this diversity–ability battle (see Figures 1–3). In environments where good cues are very good and bad cues are quite bad, having a group of agents who can use this disparity properly and as a result achieve higher individual accuracy is more important than having another group of agents who know little and can only rely on diversity to boost their group's performance; in other environments where cues are close in the quality of their information, the reverse tends to be true. Moreover, we found that task environment strongly moderates the effects of group size (Figure 1) and individual learning (Figure 2), but to a much lesser extent the effect of information errors (Figure 3).

Information is critical to the survival of an organism in the environment that is not always reliable, due to the instability of the environment (e.g., bad weathers) and the limited information processing ability of the organism (e.g., in perception and memory). Erroneous information, as shown in our Study 3, will reduce the decision accuracy of an individual and possibly its fitness if decisions are assumed to be consequential. However, this obstacle can be easily overcome by a group of individuals, so much so that a group is even able to achieve a higher level of accuracy with erroneous information than with error-free information. The robustness of groups against errors has been documented [3], [23], [31]; however, to the best of our knowledge, ours is the first study demonstrating this *overcompensation* effect of erroneous information. The effect shows how “flawed” individuals may rely on groups to achieve feats that cannot be reached by each of them, and implies that groups should be tolerant towards, even welcoming, the occasional errors made by its members.

Finally, a frequent feature of group decision-making is the use of public information by group members [25], [26], [36]. In our study, public information can be conveyed as either the actual decisions made by other members (e.g., flowers other bees choose to forage) or the cue orders they adopt to reach their decisions. It has been shown that such information can be beneficial to the accuracy of individual members who apply heuristics such as take-the-best and minimalist to make decisions, but only to a limited extent to their groups [37], [38]. A likely effect of public information is the convergence of members' knowledge and decisions, which, as demonstrated in both our Study 2 and other studies [4], [6], [26], will reduce group diversity and may be detrimental to the accuracy of group decisions and judgments. From our perspective, public information is a double-edged sword like learning and information errors, because of its conflicting effects on individual accuracy and group diversity. To understand more precisely how it affects the overall group performance could be a meaningful topic for future research.

## Methods and Analyses

### Take-the-Best and Minimalist

Take-the-best is composed of three rules that cover the processes of searching, stopping search, and deciding: (1) It searches cues sequentially in the order of their validities (defined below); (2) assuming that cues are expressed in or can be converted to binary values, search stops whenever two options have different values on a cue (i.e., [1, 0] or [0, 1]); and (3) a decision is made according to the options' values on the cue that stops search, with the option having a higher cue value usually inferred as having a larger criterion value. If all the cues are searched and none of them differ in their values on the two options, a decision will be made through random guessing. The rules of minimalist are similar to those of take-the-best, with one exception: Instead of searching by their validity order, minimalist searches cues randomly. This makes minimalist generally less accurate than take-the-best, and the larger the differences among cues' validities, the larger the accuracy gap between the two heuristics [13].

A cue's validity *V* is calculated by *V* = *R*/(*R*+*W*), where *R* and *W* are the number of correct (right) and incorrect (wrong) decisions, respectively, made by using that cue when two options have different values on it. The measure of validity is mathematically related to the Goodman-Kruskal Gamma coefficient that has been used in a broad range of tasks [39]. A cue's objective validity is defined as its validity in a population of options, which may differ from its validity derived from a sample. In addition, if a cue is positively correlated with the criterion, *V* should have a value larger than 0.5. When *V* is less than 0.5, it implies that the decision rule should be used in the reverse way.

### Simulation Architecture

All of our simulations were programed and run in Matlab. To simulate the task studied here, we need to generate one criterion variable and several cue variables correlated with it, such that each cue can carry some diagnostic value. In the case of honeybee foraging, for example, the criterion variable may be the attractiveness of a flower that correlates to certain extents with the odor of the flower, the particle size of its pollens, and the handling time required for extraction [40]. A popular way to generate these variables in simulations is to create a criterion that is the linear combination of the cues and a random error component [41]. Five independent cues were first generated in our simulations and then combined to create the criterion variable using the following equation:
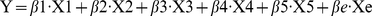
In the equation, *Y* is the criterion variable, *X_i_* is a cue variable with a standardized normal distribution N(0,1), and β*_i_* is the linear coefficient of each cue variable *X_i_*. *X*_e_ is the error term, which also has an N(0,1) distribution, and β_e_ is its linear coefficient. The number of cues was set at five because pilot simulations showed that more than three cues were needed to achieve a high enough level of diversity for the minimalist group and the probability of searching beyond five cues by either take-the-best or minimalist was very small.

Using this general equation, we created four task environments, each characterized by a different set of βs; their specific values can be seen in Table 1. We called the first one the large-difference (LD) environment, for there was a large variance among the cues' linear coefficients. The variance decreased gradually from the LD to the medium-difference (MD), the small-difference (SD), and finally the no-difference (ND) environment, where cue coefficients were equal. Despite the apparent differences in the four environments, they did have one thing in common: Cues in each environment could together account for almost the same proportion of variance in the criterion variable.

**Table 1:** The Linear Coefficients (β) and Validities (*V*) of the Five Cues in Four Task Environments.

Environment	β_1_, β_2_, β_3_, β_4_, β_5_, β_e_	*V*_1_, *V*_2_, *V*_3_, *V*_4_, *V*_5_	Variance accounted for by the cues (%)
Large difference	0.37, 0.23, 0.11, 0.07, 0.04, 0.18	0.86, 0.71, 0.60, 0.57, 0.54	0.865
Medium difference	0.26, 0.20, 0.16, 0.13, 0.11, 0.16	0.78, 0.71, 0.67, 0.64, 0.61	0.864
Small difference	0.19, 0.18, 0.17, 0.16, 0.15, 0.15	0.71, 0.70, 0.69, 0.68, 0.67	0.866
No difference	0.17, 0.17, 0.17, 0.17, 0.17, 0.15	0.69, 0.69, 0.69, 0.69, 0.69	0.865

Because take-the-best and minimalist use binary cues to make decisions, it is necessary to dichotomize the cues. We used the median of a cue's value distribution as the cutoff, above which a cue value was converted to 1 and below which to 0. All cue validities were then calculated (see Table 1). As the table shows, the larger the β, the higher the validity in an environment. Therefore, the differences in cues' βs were transferred directly to the differences in their validities. As a result, there was a highly dispersed distribution of cue validities in the LD environment and a totally flat distribution in the ND environment, with the MD and SD environments in between.

With a continuous criterion variable and five binary cues (after dichotomization) making up our simulated data, we took the following general steps to obtain a group's decision accuracy in a certain task environment:

Step 1: Draw a random sample of data with *n* options in it.
Step 2: For any pair of options in the sample, implement the rules of either take-the-best or minimalist to make an individual decision for an agent.
Step 3: Repeat Step 2 until all *m* agents in a group make their decisions.
Step 4: Apply the simple-majority rule to make a group decision.
Step 5: Repeat Steps 2–4 until all pairs of options in the sample are compared.
Step 6: Calculate both the agents' and their groups' decision accuracy within a sample based on options' actual criterion values.
Step 7: Repeat Steps 1–6 with *N* random samples and use the means of the results from all samples as the final results.

### Specific Simulation Procedures

In Study 1 (group size), there were 15 options (*n* = 15), which produced 105 pairs of options (15×14/2) in each random sample. Four group sizes were examined: *m* = 1, 5, 15, and 100; and for each group size, 10,000 samples were run to get the results.

In Study 2 (individual learning), there were two types of samples in each run. The first was the learning sample, in which a take-the-best agent calculated each cue's validity based on all possible pairs of options in the sample. For a group of *m* agents, *m* random samples were drawn for learning, one for each agent. Then, there was the testing sample. Unlike the learning sample, there was only one testing sample for a group. All agents would use the cue validity orders they had learned previously to guide their search and decision-making in this common sample. The size of the learning sample, as measured by the number of options in the sample *n*, was the parameter manipulated in this study. Five levels were tested: 10, 15, 25, 50, and population. In all conditions, the size of the group was set at 5, each testing sample consisted of 15 objects, and 10,000 random samples were run for both learning and testing.

In Study 3 (information errors), errors in cues' binary values were created by adding a certain amount of random errors to cues' continuous values before dichotomization. These errors conformed to an N(0, σ) distribution, with the parameter σ varied to control the error magnitude. At a given error level, an independent set of random errors was added to each cue and for each group member. Hence, for *i* cues and *m* members, a total of i*m sets of random errors were generated. Five values of σ were applied; and the larger the σ, the more likely that a cue's binary values would flip. In all conditions, the group size was set at 5, the number of objects in a task sample was 15, and 10,000 random samples were run to get the results.

## Supporting Information

File S1 **Diversity Results.** The results are shown in three sheets in the Excel file, corresponding to the results in Study 1, 2, and 3, respectively.
(XLS)
